# Adaptation of a Cyanobacterium to a Biochemically Rich Environment in Experimental Evolution as an Initial Step toward a Chloroplast-Like State

**DOI:** 10.1371/journal.pone.0098337

**Published:** 2014-05-29

**Authors:** Kazufumi Hosoda, Masumi Habuchi, Shingo Suzuki, Mikako Miyazaki, Go Takikawa, Takahiro Sakurai, Akiko Kashiwagi, Makoto Sueyoshi, Yusuke Matsumoto, Ayako Kiuchi, Kotaro Mori, Tetsuya Yomo

**Affiliations:** 1 Institute for Academic Initiatives, Osaka University, Suita, Osaka, Japan; 2 Department of Frontier Biosciences, Graduate School of Frontier Biosciences, Osaka University, Suita, Osaka, Japan; 3 Quantitative Biology Center, RIKEN, Suita, Osaka, Japan; 4 Department of Biotechnology, Graduate School of Engineering, Osaka University, Suita, Osaka, Japan; 5 Department of Bioinformatic Engineering, Graduate School of Information Science and Technology, Osaka University, Suita, Osaka, Japan; 6 Faculty of Agriculture and Life Science, Hirosaki University, Hirosaki, Aomori, Japan; 7 Exploratory Research for Advanced Technology (ERATO), Japan Science and Technology Agency, Suita, Osaka, Japan; University of Florida, United States of America

## Abstract

Chloroplasts originated from cyanobacteria through endosymbiosis. The original cyanobacterial endosymbiont evolved to adapt to the biochemically rich intracellular environment of the host cell while maintaining its photosynthetic function; however, no such process has been experimentally demonstrated. Here, we show the adaptation of a model cyanobacterium, *Synechocystis* sp. PCC 6803, to a biochemically rich environment by experimental evolution. *Synechocystis* sp. PCC 6803 does not grow in a biochemically rich, chemically defined medium because several amino acids are toxic to the cells at approximately 1 mM. We cultured the cyanobacteria in media with the toxic amino acids at 0.1 mM, then serially transferred the culture, gradually increasing the concentration of the toxic amino acids. The cells evolved to show approximately the same specific growth rate in media with 0 and 1 mM of the toxic amino acid in approximately 84 generations and evolved to grow faster in the media with 1 mM than in the media with 0 mM in approximately 181 generations. We did not detect a statistically significant decrease in the autotrophic growth of the evolved strain in an inorganic medium, indicating the maintenance of the photosynthetic function. Whole-genome resequencing revealed changes in the genes related to the cell membrane and the carboxysome. Moreover, we quantitatively analyzed the evolutionary changes by using simple mathematical models, which evaluated the evolution as an increase in the half-maximal inhibitory concentration (IC_50_) and estimated quantitative characteristics of the evolutionary process. Our results clearly demonstrate not only the potential of a model cyanobacterium to adapt to a biochemically rich environment without a significant decrease in photosynthetic function but also the properties of its evolutionary process, which sheds light of the evolution of chloroplasts at the initial stage.

## Introduction

Chloroplasts originated from cyanobacteria through endosymbiosis [1]–[4]. Chloroplasts conduct photosynthesis and synthesize most fatty acids and some amino acids and hormones [5]. Although chloroplasts play many important roles in plant cells, they lack many genes and functions compared to cyanobacteria, depending instead on the host plant cells. These data suggest that the original cyanobacterial endosymbiont adapted to the environment in plant cells, which is biochemically richer than the environment of free-living cyanobacteria [6]. Thus, the original cyanobacterial endosymbiont evolved to depend on biochemical substances provided by the host plant cell, maintaining their photosynthetic functions to provide that cell with biochemical substances in return.

It is not clear how cyanobacteria can adapt to a biochemically rich environment while maintaining their photosynthetic function. Cyanobacteria inhabit almost every niche on earth, including freshwater and salty, hot, and arid environments [6], and employ various stress-management mechanisms [7]. Cyanobacteria also establish long-lived symbiotic associations with fungi and within the plant kingdom [8]; however, cyanobacteria are sensitive to many important biochemical substances. For example, in the case of *Synechocystis* sp. PCC 6803, a prominent model cyanobacteria, the original strain (stocked in the Pasteur Culture Collection) is sensitive to glucose in light [9]. While glucose-tolerant lines exist, they are still sensitive to several amino acids [10]–[12], possibly because their autotrophic ability opposes the ability to use nutrient biochemical substances. Indeed, Bell and Reboud [13]–[15] experimentally evolved *Chlamydomonas reinhardtii* in light (photoautotrophic) and dark (heterotrophic) environments and showed that some lines in the dark environment evolved to have higher specific growth rates in the dark environment but lower specific growth rates in the light environment, although *C. reinhardtii* is not a cyanobacterium. The experimental demonstration showing that cyanobacteria adapt to a biochemically rich environment sheds light on the mechanism of adaptation and how cyanobacteria evolved to become chloroplasts.

In this study, we experimentally evolved *Synechocystis* sp. PCC 6803 to adapt to a biochemically rich environment without loss of autotrophic ability. We used a synthetic medium (TCM1) that is sufficiently nutrient-rich to allow the growth of a heterotrophic model protozoan, *Tetrahymena thermophila,* and to inhibit growth of the cyanobacteria. We first identified the six amino acids in TCM1 that are toxic to the cyanobacteria (Figure S1 in File S1). We then cultivated the cyanobacteria in the medium with reduced concentrations of the toxic amino acids and serially transferred the culture, gradually increasing the amino acids concentrations. We found that the cyanobacterium adapted to the rich environment in approximately 100 generations, and we detected no significant difference in autotrophic growth in an inorganic medium between the ancestral and evolved populations. Whole-genome resequencing revealed changes in genes related to the cell membrane and to the carboxysome, which is involved in photosynthesis. Our results clearly show that the model cyanobacterium can evolve to adapt to a rich environment with no significant decrease in photosynthetic function and experimentally show the properties of the evolutionary process of the cyanobacteria to adapt to a biochemically rich environment.

## Materials and Methods

### Strains and culture conditions

The *Synechocystis* sp. PCC 6803 (GT) strain, originally obtained from Dr. J. G. K. Williams (Dupont de Nemours, Wilmington, DE, U.S.A.), was used as the ancestral strain. This is a glucose-tolerant strain and not the original strain (stocked in the Pasteur Culture Collection). All cells were grown at 30°C in 10 mL liquid culture in a 50 mL Erlenmeyer flask rotated at 80 rpm using NR-3 (Taitec, Saitama, Japan), with 10 µmol·m^−2^·s^−1^ light using an LED system (MIL-C1000T, MIL-U200, MIL-R18A, and MIL-B18A; Sanyo, Osaka, Japan). The LED board ratio of red (MIL-R18A) to blue (MIL-B18A) was 9. We used TCM1, TCM0, BG-11, and BG-11+AA for the culture media. TCM1 was derived from a chemically defined medium for a heterotrophic model protozoan, *Tetrahymena thermophila* CDM15 [16], with a few modifications (see Table S1 in File S1 for the detailed components). TCM1 includes the following 6 amino acids that are toxic to cyanobacteria: Arg, His, Lys, Met, Phe, and Thr in concentrations of approximately 1 mM. TCM0 does not include the 6 toxic amino acids and the other components are the same as the components in TCM1. We also used TCM*x*, where *x* is the concentration of the 6 toxic amino acids relative to TCM1 (*e.g.*, TCM0.1 when *x* = 0.1). BG-11 is an inorganic medium that is standard for *Synechocystis* sp. PCC 6803 culture [17]. BG-11+AA includes the 6 toxic amino acids in the same concentrations as the TCM1 and the other components are the same as BG-11.

### Evolution Experiment

**Figure 1:**
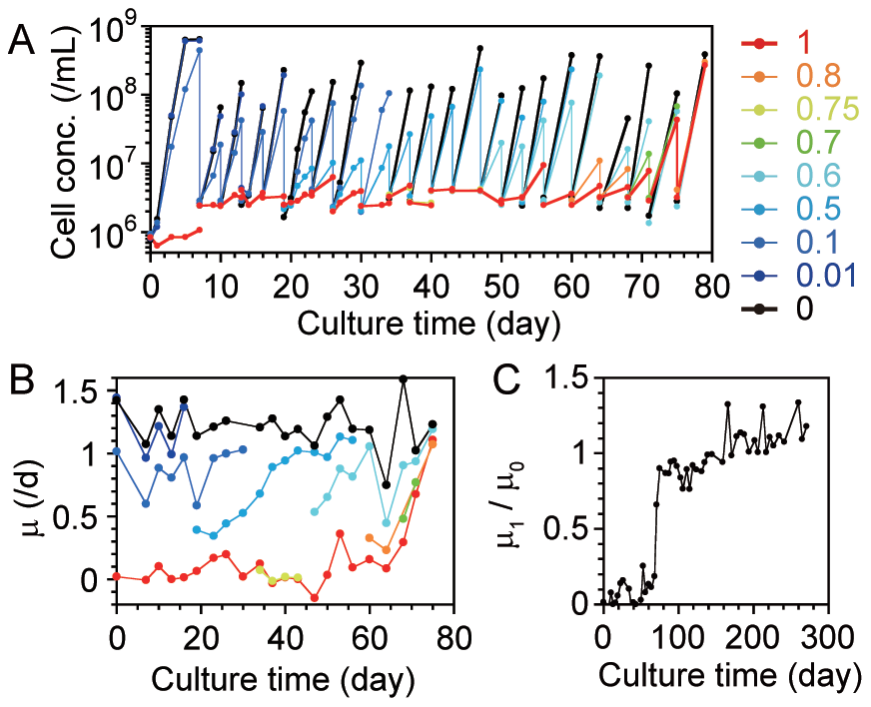
Evolutionary changes during adaptation to a nutrient-rich environment. We cultivated the cells in media with various concentrations of toxic amino acids until culture day 79, then used only TCM1 and TCM0. (A) Population dynamics in the evolution experiment until day 79. The colors show the culture media used (TCM*x* media; *x* is shown on the right). The transfers of the culture by dilution are depicted as the vertical decrease in the cell concentration. (B) Evolutionary changes in the specific growth rate (*μ*) in media with different toxic amino acid concentrations (the colors are same as in A). The specific growth rate was determined by the slope of the linear regression of the natural log of cell concentration per day, using first three (or two, if only two points were measured in the culture) data points. (C) The evolutionary changes in the relative specific growth rates of the cells in TCM1 (*μ*_1_) and in TCM0 (*μ*_0_). The raw data of the population dynamics are shown in Figure S4 in File S1.

We first cultivated the cells (pre-cultured in BG-11) in TCM0, transferred them 4 times (as an initial adaptation, see Figure S2 in File S1), freeze-stocked them once by adding DMSO to the culture (final 7%), and recovered them in TCM0 before beginning the experimental evolution. The culture conditions were the same as in the evolution experiments except for the culture media. Simply, the difference between BG-11 and TCM0 is that TCM0 includes amino acids, vitamin, nucleosides, and glucose but BG-11 does not. We then serially transferred the cell culture following the protocol described below, always under 10 µmol·m^−2^·s^−1^ of light. At every transfer until day 79, we split the cell culture into three or four TCM*x* media that differed in *x*. After the cultivation, we identified the culture with the highest *x* that showed a population increase of more than the square root of 10, and we used it as the origin for the next transfer. The initial cell concentration of each transfer was set to be approximately 2×10^6^ cells/mL (thus the dilution rate for transfers was different for each round and the bottleneck size of the population was 2×10^7^ cells in 10 mL), and the actual cell concentrations in the evolution experiment are plotted in Figure 1A. The incubation time for each culture was 3 or 4 days so that we maintained almost exponential phase for the transferred culture (under approximately 2×10^8^ cells/mL) as semi-continuous culture. After day 79, we serially transferred the cell culture to TCM1, examining the growth in TCM0 in parallel until day 271 (see Figure S4 in File S1). The generation during evolution was calculated as ln(Δ_rpop_)/ln2, where Δ_rpop_ is the total fold change in the population.

### Measurement of cell concentrations

We measured the cell concentrations relative to a known concentration of fluorescent beads (Fluoresbrite YG Microspheres, 6 µm; Polysciences Inc., Warrington, PA, USA) using a Cytomics TM FC500 flow cytometer (Beckman Coulter, Inc., CA, USA) by loading culture samples mixed with the beads as described previously [18]. A 488-nm argon excitation laser was employed, and bandpass filters of 515–535 and 610–630 nm were used to measure green and red fluorescence, respectively. The cyanobacterial cells were detected as red fluorescent particles due to the presence of chlorophyll, and the cell concentration was calculated from the counts of these particles relative to the fluorescent beads, which were detected as green fluorescent particles.

### Whole-genome resequencing

We analyzed 3 genome sequences of (i) ancestral cells, (ii) initially adaptive cells in TCM0 (Figure S2 in File S1), and (iii) evolved cells (at 408 days, 85 transfers, 548 generations, in TCM1). We prepared precultures by shaking stocked populations in media of BG-11, TCM0, and TCM1 for (i), (ii), and (iii), respectively, under the same culture conditions as described above, until the cell concentrations reached approximately 1×10^8^ cells/mL. The cyanobacterial cells were collected by centrifugation at 14,170×*g* for 2 min at 25°C, and the pelleted cells were stored at −80°C prior to genomic DNA purification. Genomic DNA was isolated and purified in accordance with a standard method [19]. Genome sequence analyses were performed with an Illumina MiSeq sequencer (Illumina, CA, USA) by Eurofins MWG Operon (AL, USA). Three hundred basepair insert libraries were generated according the Illumina protocol and sequenced in an Illumina MiSeq. In this study, 3 samples with different indexes were mixed and then sequenced in 2×150 bp sequencing mode. We obtained 1079, 917, and 1110 Mbp sequences for (i), (ii), and (iii), respectively. These numbers of base calls corresponded to ×301.9, ×256.6, and ×310.6 redundancy, respectively. We used Illumina's CASAVA pipeline version 1.8.2 software (Illumina, CA, USA) for data analyses including trimming and alignment to the reference genome sequence NC_000911.1 (*Synechocystis* sp. PCC 6803 chromosome, complete genome). We used VarScan 2 software [20] for variant analysis.

### Modeling and simulations

Simulations of the evolutionary change in ln[IC_50_] were performed using Matlab software (MathWorks, MA, USA). The Matlab code used is presented in Appendix S1 in File S1.

## Results and Discussion

### Evolutionary changes in adaptation to a nutrient-rich environment

We serially transferred the cyanobacterial cell culture, gradually increasing the concentrations of the 6 amino acids that were toxic to the cells and always maintaining the cultures under light to maintain their photosynthetic function. Figure 1A shows cell growth during evolution; the cells evolved the ability to grow in media with higher concentrations of the toxic amino acids through serial transfer. Figure 1B shows the specific growth rates (*μ*) in media with different toxic amino acid concentrations, TCM*x*, where *x* is the relative amino acid concentration (plots of *μ* as a function of *x* are shown in Figure S3 in File S1). We found a statistically significant increase in *μ* in TCM1 (*μ*_1_, red line) while we did not find statistically significant increases in *μ* in TCM0 (*μ*_0_, black line) by a Student's *t*-test to compare the slope of the linear regression of *μ* on the culture time with zero slope using a significance level of 0.05; the slopes were 0.0069 (*R* ^2^ = 0.32, the degree of freedom *DF* = 20, *p* = 0.0057) and −0.0014 (*R* ^2^ = 0.032, *DF* = 19, *p* = 0.44)/day^2^ for TCM1 and TCM0, respectively. *μ* in TCM*x* for 0<*x*<1 (colored lines) were in between *μ*_1_ and *μ*_0_. These results suggest that the key change was related not to global growth but to tolerance of the toxic amino acids. Then, we continued the transfer in TCM1 (the population dynamics are shown in Figure S4 in File S1). Figure 1C shows the evolutionary changes in *μ*_1_ relative to *μ*_0_. *μ*_1_ approached 90% of *μ*_0_ at approximately 75 days (21 transfers, 84 generations) and exceeded *μ*_0_ at approximately 160 days (40 transfers, 181 generations). These results indicate that the model cyanobacteria had adapted to a biologically rich environment, increasing their tolerance to the previously toxic amino acids.

### Autotrophic ability of the evolved strain

**Figure 2:**
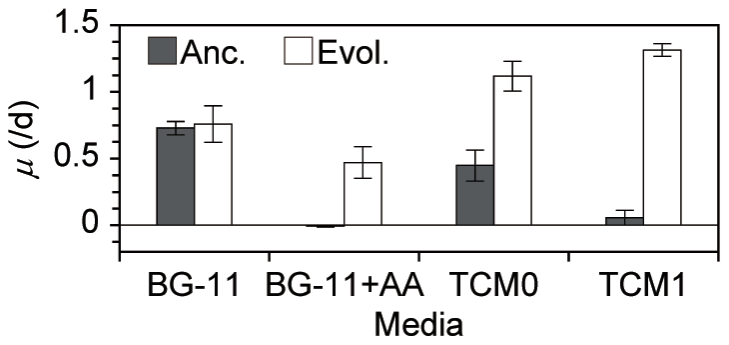
Specific growth rates of the ancestral and evolved cells in various environments. The black and white bars indicate the specific growth rates of the ancestral and evolved (at 408 days, 85 transfers, 548 generations) cells, respectively, in the culture media shown at the bottom. The error bars show the SD of five (for BG-11 and TCM1) or two (for the other media) independent cultures. The ancestral culture in BG11+AA showed almost zero growth.

We found no statistically significant decrease in autotrophic ability (photosynthetic function) in the evolved cells. Figure 2 shows the *μ* of the ancestral and evolved cells in various culture media. First of all, we found that the evolved cells actually increased their growth ability in TCM1 because there was a statistically significant difference in the mean *μ* in TCM1 between the evolved and the ancestral cells as determined by a Welch's *t*-test using a significance level of 0.05 (*DF* = 7, *p* = 1.9×10^−9^). Importantly, there were no statistically significant differences between the evolved and the ancestral cells in the inorganic medium BG-11 (*DF* = 5, *p* = 0.67), suggesting that the photosynthetic function was maintained in the evolved cells. Furthermore, the evolved cells showed amino acid tolerance even in BG-11 (there were no statistically significant differences between BG-11 and BG-11+AA; *DF* = 2, *p* = 0.11), while the ancestral strain was sensitive (there was a statistically significant difference between BG-11 and BG-11+AA; *DF* = 4, *p* = 5.9×10^−6^). We did not observe decreased means of the specific growth rates in the evolved cells (trade-offs) in any of the tested media.

### Whole-genome resequencing

The evolved changes in phenotypes were corroborated by the genetic changes detected by whole-genome resequencing. We analyzed 3 genomic DNA samples of ancestral cells, initially adaptive cells in TCM0 (Figure S2 in File S1), and evolved cells in TCM1. All mutations in the chromosomes found in the variant analysis are listed in Table S2 in File S1. We found 20 single-base substitutions and 8 small indels in ancestral cells compared with the reference sequence (NC_000911.1 *Synechocystis* sp. PCC 6803 chromosome, complete genome). These mutations were also detected in the other two types (initially adaptive and evolved) of cells. The detected mutations were identical for the ancestral and initially adaptive cells (see Table S2 in File S1).

The mutations detected only in the evolved strains are summarized in Table 1. All of the single-base substitutions identified in annotated genes were non-synonymous, suggesting that they were adaptive. Mutations in genes related to membrane biogenesis and transportations were detected (#3, 5 and 6 in Table 1), suggesting that the amino acid tolerance arose from changes in membrane functions, such as increased membrane protection from penetration by amino acids. Although gene #4 was annotated as an unknown protein, it has been reported that this gene is related to stress responses, such as butanol, hexane, salt stresses and nitrogen starvation [21]. These results are consistent with the increase in tolerance. A mutation in the carboxysome-formation protein CcmA [22] (#1) might indicate an optimization of photosynthetic activity in a biochemically rich environment because the CO_2_-concentration mechanism (CCM) of the carboxysome is important to meeting habitat requirements [7]. A mutation was also identified in the RNA polymerase alpha subunit (#2). Although it is difficult to estimate the direct involvement of this mutation in the evolved changes in the phenotypes, the mutation in the RNA polymerase alpha subunit could potentially provide some benefit because the initiation of transcription is the main determinant for the regulation of gene expression in bacteria. Note that those individual mutations would not be directly correlated with acquired phenotype such as by the existence of epistasis [23], [24] and epigenetic changes [25]. Further work, including construction of a mutant containing these mutations and analysis of phenotypes of these mutants, is needed to elucidate the relationship between genotype and phenotype. Also note that we analyzed mixed (not cloned) populations that were stocked directly from the evolution experiment, and some heterogeneous mutations were found (see Table S2 in File S1). However, it is possible that this heterogeneity did not arise from the heterogeneity of different cell groups because it is known that *Synechocystis* sp. PCC 6803 has a multicopy genome [26], and heterozygous mutations are commonly found in the genome of the *Synechocystis* sp. PCC 6803 [27]. Actually, the heterogeneic frequencies were maintained during the course of evolution, and the specific mutations in evolved cells shown in Table 1 were almost homogeneous (see Table S2 in File S1).

**Table 1:** Genetic changes detected in the evolved cells relative to the ancestral cells.

#	Position (nt)	Nucleotide changes	Gene	AA changes in the Gene
1	335496	C to A	Carboxysome-formation protein CcmA; complementary	G to E
2	829508	C to T	RNA polymerase alpha subunit; complementary	R to Q
3	1114921	G to C	Periplasmic substrate-binding and integral membrane protein of the ABC-type Bgt permease for basic amino acids and glutamine BgtB; complementary	F to L
4	1128135	C to G	Unknown protein (sll1265); complementary	A to P
5	Next to 1905171	Del. GCCTCG	Penicillin-binding protein (cell-wall biogenesis); complementary	Del. AE
6	3203715	G to A	Probable cation transporter; complementary	P to S

All detected mutations in the chromosome of the ancestral cells, initially adaptive cells, and the evolved cells, relative to the reference (NC_000911.1; Synechocystis sp. PCC 6803 chromosome, complete genome), are shown in Table S2 in File S1.

### Quantitative analyses of the evolutionary changes using simple mathematical models

**Figure 3:**
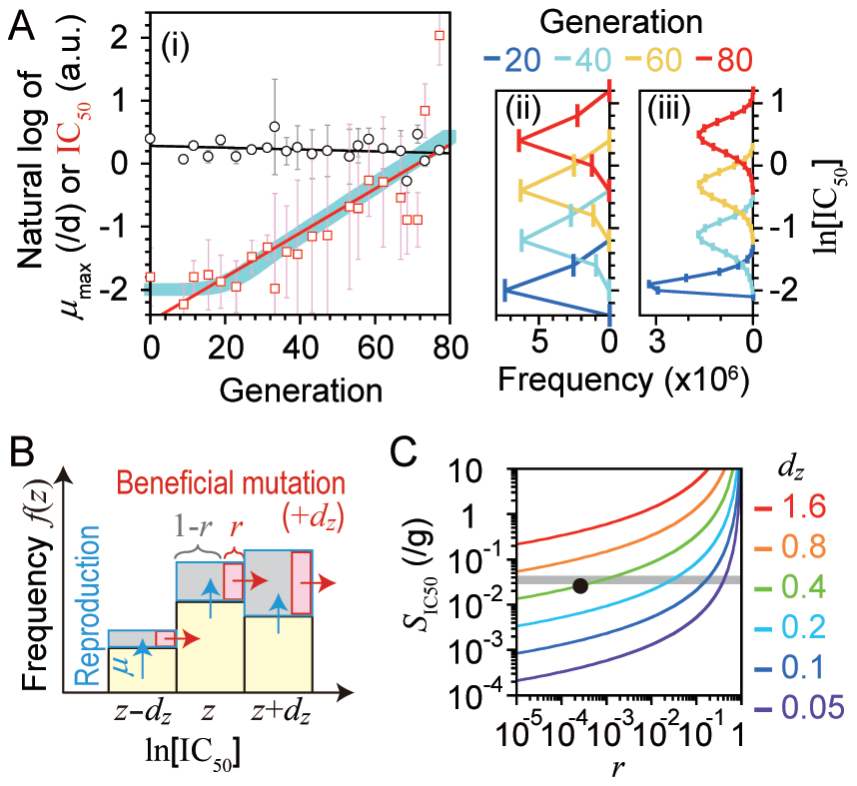
Quantitative analyses of the evolutionary changes by simple mathematical models. (A) Evaluation of the evolutionary changes in the maximum specific growth rate (*µm*_ax_) and the half-maximal inhibitory concentration (IC_50_) until day 79. (i) Evolutionary changes in ln[*µm*_ax_] (black circle) and ln[IC_50_] (red square), determined by the fitting shown in Fure S3 in File S1, with respect to the generation (*g*) of each round. The error bars show the standard error of the fitting estimates. The black and red lines show the linear regression of ln[*µm*_ax_] and ln[IC_50_], respectively (−0.0014*g*+0.28 and 0.035*g*–2.5, respectively). The blue bold line is the change in the mean of ln[IC_50_] calculated by the numerical simulation of the mathematical model of Eq. 1, using (*d_z_*, *r*, *N*) = (0.4, 0.00023, 10^7^), assuming a homogeneous population for the initial state. The source code of the simulation is shown in Appendix S1 in File S1. (ii and iii) Simulated evolutionary changes in frequency distributions of the cell population calculated by the numerical simulation using (*d_z_*, *r*, *N*) of (0.4, 0.00023, 10^7^: the same as the blue line in A-i) and (0.1, 0.04, 10^7^), respectively. The vertical axis is ln[IC_50_], as in A-i. (B) A conceptual scheme of the mathematical model of Eq. 1. See the text for explanation. Simply, the population shifts toward greater ln[IC_50_] with a beneficial mutation rate per generation (*r*) and an effect size of a single beneficial mutation on ln[IC_50_] (*d_z_*). The axes correspond to those of A-ii and A-iii. (C) The dependency of the evolutionary rate of ln[IC_50_], *i.e.*, the slope of the red line in A-i (*S*_IC50_), on the model parameters *d_z_* and *r* calculated from Eq. 2 using *N* = 10^7^. The gray bold line shows the experimentally determined value (*S*_IC50_ = 0.035/*g*). The black point shows the value used for the simulation shown in A-ii, which is discussed in the text.

We analyzed the experimental results of the evolutionary changes using simple mathematical models to extract the quantitative properties of the evolution. Specifically, we estimated a key trait (IC_50_, see below) in the evolution, the trait variance in the population, and some properties of the fitness landscape regarding the key trait, such as the probability of the beneficial mutation per replication and the extent of the change in the key trait per beneficial mutation. We used the data through day 75 (77 generations) where the growth rate of the cells in the transferred cultures was less than *μ*_0_. First, we estimated the maximum specific growth rate (*µm*_ax_) and the half-maximal inhibitory concentration (IC_50_) of the cell population for every transfer round using the experimental data of the relationship between the specific growth rate and the amino acid concentration (Figure 1B, and Figure S3 in File S1). Specifically, we estimated those two parameters by fitting the experimental data to a basic noncompetitive-inhibition model *μ* = *µm*_ax_/(1+*x*/IC_50_) [28] (the fitting results are shown in Figure S3 in File S1). Figure 3A-i summarizes the fitting results and clearly shows that the principal increase was in IC_50_, not *µm*_ax_; there was a statistically significant increase in ln[IC_50_] (red square) while not in ln[*µm*_ax_] (black circle) as determined by a Student's *t*-test to compare the slope of the linear regression of the parameters on generation with zero slope using a significance level of 0.05; the slope of ln[IC_50_] and ln[*µm*_ax_] was 0.035 (*R* ^2^ = 0.67, *DF* = 20, *p* = 3.6×10^−6^) and −0.0014 (*R* ^2^ = 0.037, *DF* = 20, *p* = 0.39) per generation, respectively. Thus, it is possible to quantitatively understand this evolutionary change as the change in a trait, IC_50_, based on quantitative genetics as described below.

We analyzed the evolutionary rate of the IC_50_, specifically the slope of ln[IC_50_] (designated as *S*_IC50_), to provide an interpretation of the experimental results, using a simple model of the directional selection based on quantitative genetics [29]–[32]. Let us assume a cell population in which each cell has a trait *z* ( = ln[IC_50_]) and a specific growth rate *μ*(*z*), with the frequency *f*(*z*,*t*) at the total *N* (*i.e.*, ∑*f*(*z*,*t*) = *N*). When a cell with *z* reproduces an offspring through replication, the trait of the progeny becomes *z*+*d_z_* by a beneficial mutation with a constant probability *r*, or else keeps *z* (probability 1−*r*), which is the same as the parent (Figure 3B), *i.e.*, *r* is a beneficial mutation rate per generation and *d_z_* is an effect size of a single beneficial mutation on the trait *z*. Constant *r* and *d_z_* indicate that this model assumes a non-epistatic landscape [33]. The rate equation of *f*(*z*,*t*) is described as
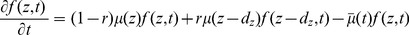
(1)where 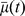 is the mean specific growth rate of the population (see Text S1 in File S1 for the derivation). The first and second terms on the right-hand side represent the increase in the population frequency of *z* by reproduction from the population of *z* without any beneficial mutation (a gray square fraction at *z* in Figure 3B) and by reproduction from the population of *z*−*d_z_* with a beneficial mutation (a red square fraction at *z*−*d_z_* in Figure 3B), respectively. The third term represents the decrease in the population by a non-selective dilution to keep the total population size (*N*) constant. The rate of the evolutionary change in the mean of *z* over generations in this model has the same meaning as *S*_IC50_ if constant. To obtain a simple understanding of the value of *S*_IC50_, we used a set of rough approximations to obtain an approximate analytical solution of *S*_IC50_ from the model (see Text S1 in File S1). We found that the evolutionary rate became constant in this model and obtained its approximate analytical solution as
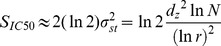
(2)where *σ_st_* is a standard deviation of the trait *z* (the width of the trait frequency distribution) when the evolutionary rate is constant. Equation 2 indicates that *S*_IC50_ is a monotonically increasing function of *d_z_*, *r*, and *N* for the ranges *d_z_*>0, 0<*r*<1, and *N*>1. The difference between this approximate analytical solution Equation 2 and the numerical simulation according to Equation 1 is shown in Figure S5 in File S1. Equation 2 means that the evolutionary rate is proportional to the variance (*σ_st_* ^2^), known as Fisher's fundamental theorem of natural selection [34], and *σ_st_*≈0.16 was obtained from the experimental value of *S*_IC50_ ( = 0.035) independently of the model parameters (see Figures 3A-ii and 3A-iii for graphical presentations of the frequency distribution).

Undetermined variables were only *d_z_* and *r* because *N* was given in the experimental condition. The dependency of *S*_IC50_ on *d_z_* and *r* is shown in Figure 3C. Therefore, if one of the two variables is determined, the other can also be determined from the experimental results, and the evolution can be visually reconstituted by the model, as shown by the blue line in Figure 3A-i and the corresponding population changes in Figure 3A-ii using (*d_z_*, *r*) = (0.4, 0.00023) as an example. This value of *d_z_* = 0.4 (*i.e.*, *S*_IC50_ increases 1.5-fold per beneficial mutation) is possible because the extent of the total increase in ln[IC_50_] that is required to satisfy *μ*_0_≈*μ*_1_ (Δ_z_) seems to be approximately 2.5 (−2 to 0.5) based on Figure 3A-i, and the total number of beneficial mutations (*n_m_*) can be 6 based on the number of detected mutations, resulting in *d_z_* = Δ_z_/*n_m_*≈0.4. Then, *r* should be approximately 0.0002 to explain the experimental results by this model with *d_z_* = 0.4 as above. With *r*≈0.0002 (*i.e.*, 0.02% of replications are beneficial), the fraction of beneficial mutation among all possible mutations can be estimated as 0.07 (*i.e.*, 7% of mutations are beneficial) from the value of *r* divided by the mutation rate per genome per replication *m*_rep_, which is known to be approximately 0.003 in microbes [35]. Note that *r* is more sensitive than *d_z_* in those range (Equation 2 and Figure 3C), *e.g.*, if the range of *d_z_* is 0.3∼0.5, the range of *r* is approximately 10^−6^∼10^−3^. Also note that above analyses are rough estimation and validity is still unclear. For example, the total number of beneficial mutations *n_m_* can be less than 6, for reasons such as non-beneficial mutations in Table 1 or few mutations until day 75. In addition, *n_m_* also can be greater than 6 because heritable trait changes can occur not only through mutations but also by other epigenetic factors [25]. Although the validity of the values and the model itself are still unclear in this study, the above information provides important insights into the evolutionary process of the cyanobacteria to adapt to a biochemically rich environment.

## Conclusions

In this study, we show that a model cyanobacterium, *Synechocystis* sp. PCC 6803, can evolve to adapt to a biochemically rich environment without statistically significant losses of autotrophic ability. Our results thus show the potential of a cyanobacterium to evolve into a chloroplast-like endosymbiont. Furthermore, we showed some properties of the evolutionary changes such as an increase in tolerance to toxic amino acids rather than the global growth rate itself, the quantitative characteristics of the evolutionary rate, which provides estimated variance of the tolerance in the population and clues regarding the evolutionary fitness landscape, and genomic analyses that identified the mutated genes and were consistent with the increase in tolerance. Additional generations would be needed to show more qualitatively distinct changes toward a chloroplast-like phenotype, such as loss of growth ability in inorganic media while maintaining photosynthetic function and optimization of photosynthesis for a biochemically rich environment. Community-level function (shown in another cyanobacterium [36]) must be a key factor for loss of functions in the evolution [37]. Therefore, ultimately, the cyanobacteria cells would have to be cultivated with or within another eukaryotic cell [38]–[40] for the complete experimental evolution toward chloroplast-like endosymbiont.

## Supporting Information

File S1 Supporting information which contains the following. Figure S1: Toxic components in TCM1. Figure S2: Preculture for the evolution experiment. Figure S3: The specific growth rates as a function of the relative concentration of the toxic amino acids in each transfer round. Figure S4: Population dynamics in the evolution experiment. Figure S5: The difference between the approximate analytical solution (Equation 2) and the numerical simulation according to Equation 1. Table S1: Components of TCM1. Table S2: All detected mutations in the chromosome, relative to the reference (NC_000911.1; Synechocystis sp. PCC 6803 chromosome, complete genome), by the genomic analysis. Text S1: The derivation of Equations 1 and 2. Appendix S1: The Matlab source code of the simulation shown in Figure 3A-i (blue bold line).
(PDF)
