# Supplementary material for: Adaptation of a Cyanobacterium to a Biochemically Rich Environment in Experimental Evolution as an Initial Step toward a Chloroplast-Like State
Source: PLoS One. 2014 May 29;9(5):e98337. doi: 10.1371/journal.pone.0098337 (PMC4038495; doi:10.1371/journal.pone.0098337)
Supplement: File S1 — Supporting information which contains the following. Figure S1: Toxic components in TCM1. Figure S2: Preculture for the evolution experiment. Figure S3: The specific growth rates as a function of the relative concentration of the toxic amino acids in each transfer round. Figure S4: Population dynamics in the evolution experiment. Figure S5: The difference between the approximate analytical solution (Equation 2) and the numerical simulation according to Equation 1. Table S1: Components of TCM1. Table S2: All detected mutations in the chromosome, relative to the reference (NC_000911.1; Synechocystis sp. PCC 6803 chromosome, complete genome), by the genomic analysis. Text S1: The derivation of Equations 1 and 2. Appendix S1: The Matlab source code of the simulation shown in Figure 3A-i (blue bold line). (PDF) [file pone.0098337.s001.pdf]

Supporting Information

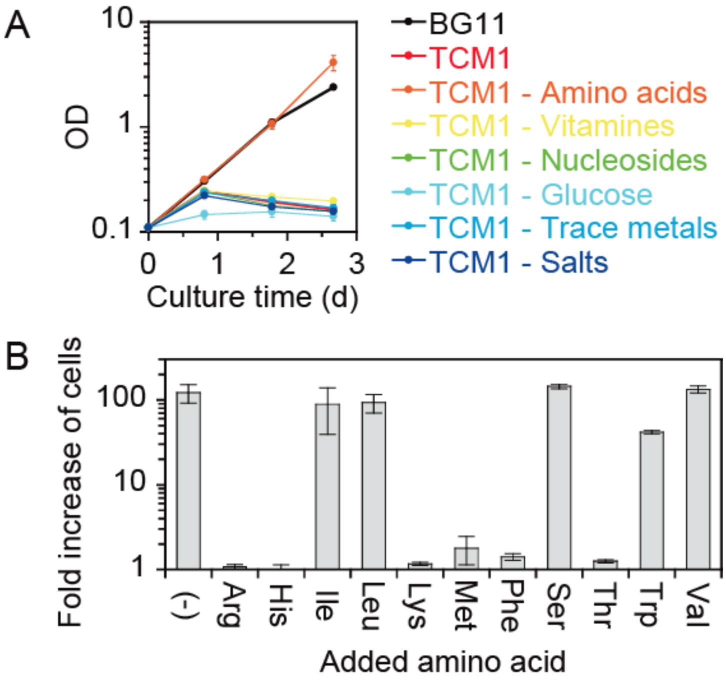

Figure S1. Components toxic to the cyanobacteria in a rich, chemically defined medium, TCM1. (A) The growth curve of the cells in the media listed to the right of the plot. Clearly, the cells grew only when the amino acids (11 different amino acids; see Table S1 for the components of TCM1) were omitted from TCM1 (except in BG-11, the positive control). (B) The *n*-fold increase in the cell concentration after 4 days of growth in media where one amino acid was added to the amino acid-omitted TCM1. The amino acid added to each culture is listed at the bottom of the plot. Cell growth was inhibited when Arg, His, Lys, Met, Phe, or Thr (but not any of the other five amino acids) was added. Thus, we identified those six as toxic amino acids.

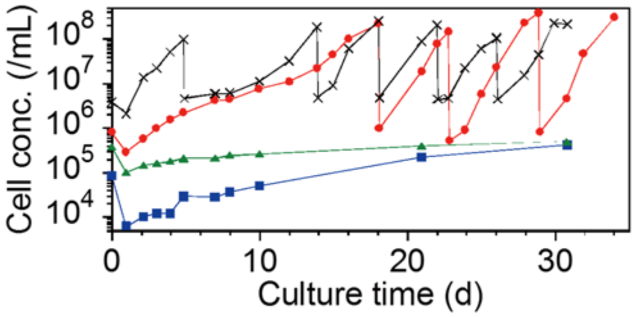

Figure S2. Preculture for the evolution experiment in TCM0. We transferred the cells from BG-11 to TCM0 and transferred them 4 times in TCM0. The colors show the cultures with different inoculation concentrations as plotted at time 0 (8.8×10^4, 3.8×10^5, 8.3×10^5, and 3.9×10^6 cells/mL for blue, green, red, and black, respectively) and we used the red line for the preculture of the evolution experiment. Growth was not stable before approximately 15 days, but later stabilized (black and red lines), showing initial adaptation from BG-11 to TCM0. We found no mutations in the genome of the initially adapted cells (red line, see Table S2).

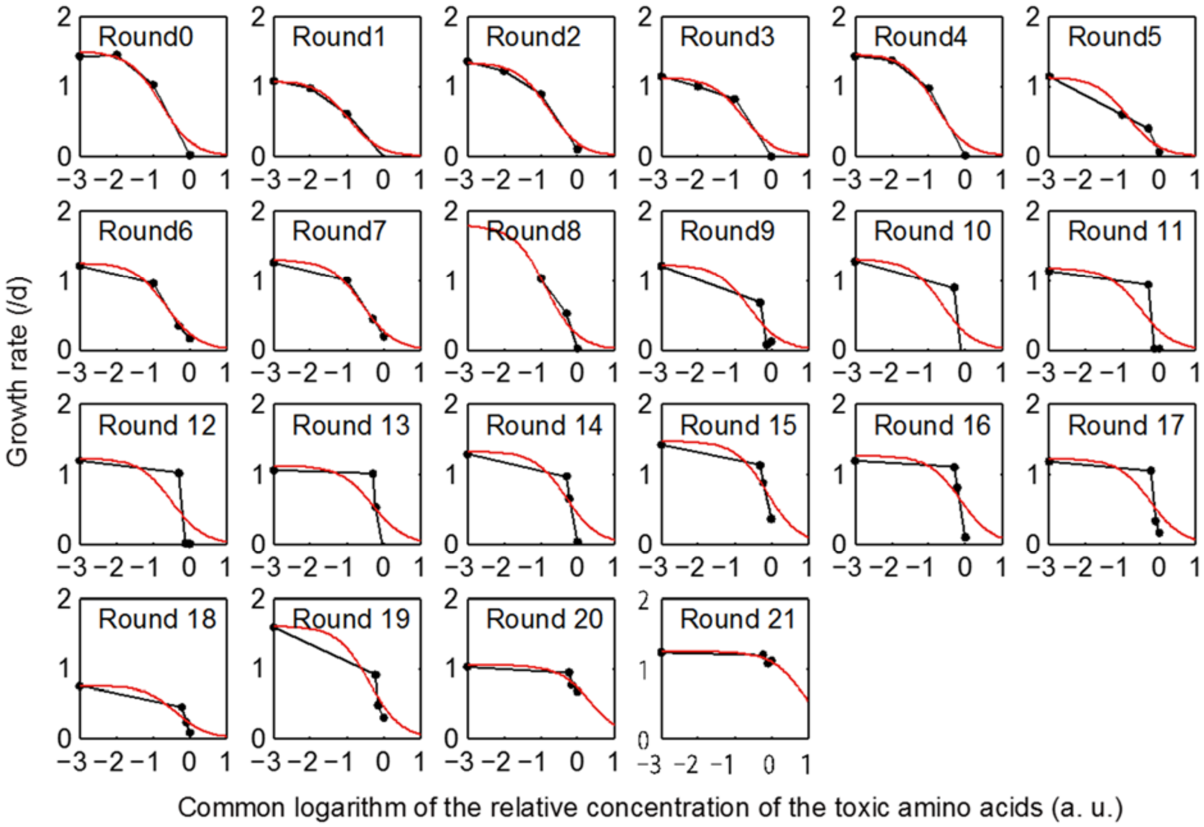

Figure S3. The specific growth rates (μ) as a function of the relative concentration of the toxic amino acids (x) in each transfer round. μ were determined from the growth curve as a slope of the linear regression of the natural log of the cell concentration when the number of data points for the round was greater than 2 and as $\ln(C_f/C_0)$ when the number of the data points for the round was 2. C_f and C_0 are the final and initial cell concentrations for the round. The red curves show the fitting of the experimental data to the equation $\mu = \mu_{max}/(1+x/IC_{50}) = e^{\beta 1}/(1+x/e^{\beta 2})$, where β1 and β2 are the fitting parameters that correspond to $\ln[\mu_{max}]$ and $\ln[IC_{50}]$, respectively. The fitting results are summarized in Figure 3A-i.

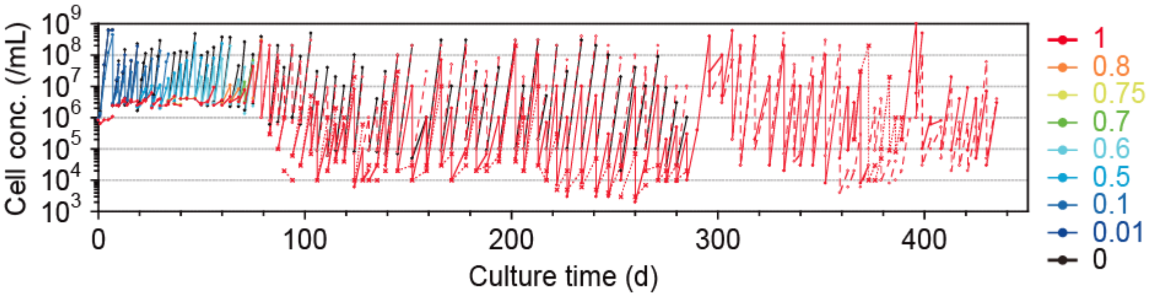

Figure S4. Population dynamics in the evolution experiment. The colors show the culture media used (TCM*x* media; *x* is shown at the right). The solid, dotted, and dashed lines are independent cultures. The transfers of the culture by dilution are shown as the vertical decrease in cell concentration. The initial cell concentration of each transfer was varied (mostly approximately 10^5 cells/mL). The cell concentration affected the growth, although the basis is still unclear. For example, less than 10^5 cells/mL seemed to make the culture unstable after day 79, and less than 10^6 cells/mL seemed to make the culture unstable at the first culture of the initial adaptation (Figure S2). Thus, we compared the growth in TCM1 to that in TCM0 at the same initial cell concentration (dashed redline and black line, respectively).

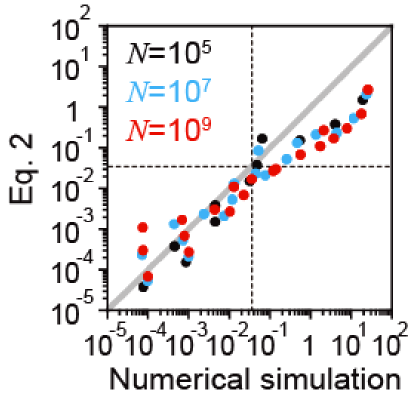

Figure S5. The difference between the approximate analytical solution of Eq. 2 and the numerical simulation according to Eq. 1. We calculated S_{IC50} by both Eq. 2 and the numerical simulation using the various parameters of r, d_z, and N. The range used for r and d_z was 10^{-5}~0.1 and 0.01~1, respectively, the same as the range shown in Figure 3C, and $N = 10^5$ (black), 10^7 (blue), or 10^9 (red). The gray solid line shows where the points should fall if Eq. 2 and the numerical simulation were equal. The dotted lines show the experimentally determined value of S_{IC50}. The deviation from the gray line becomes rather large for $S_{IC50}<0.001$ and $1<S_{IC50}$ (more than 10 fold). Because we determined S_{IC50} from the numerical simulation for generation until 100, S_{IC50} becomes too small to accurately quantify for $S_{IC50}<0.001$. For $1<S_{IC50}$, the value of the probability r used for this range was large (almost 0.1), which is out of our approximations.

Table S1. Components of TCM1. The toxic amino acids are listed in red.

Component	Concentration (mM)
**L-Arg**	1.42
**L-His**	0.95
L-Ile	1.52
L-Leu	1.52
**L-Lys**	1.09
**L-Met**	1.01
**L-Phe**	0.91
L-Ser	1.43
**L-Thr**	1.68
L-Trp	0.73
L-Val	0.85
Guanosine	7.1×10^{-2}
Uridine	8.2×10^{-2}
K_2HPO_4	1.10
KH_2PO_4	1.84
Tripotassium citrate	2.00
$MgSO_4\cdot7H_2O$	2.03
$CaCl_2$	6.8×10^{-2}
HEPES	20
EDTA·2Na	3.0×10^{-3}
H_3BO_3	4.6×10^{-2}
$NaNO_3$	17.6
Na riboflavin phosphate·$2H_2O$	9.7×10^{-4}
DL-6,8-Thioctic acid	4.8×10^{-4}
Thiamine-HCl	1.5×10^{-3}
Pyridoxal-HCl	4.9×10^{-4}
Nicotinic acid	7.3×10^{-3}
D-Pantothenic acid, Ca-salt	3.4×10^{-3}
Folinic acid, Ca-salt	2.0×10^{-4}
$FeCl_3\cdot6H_2O$	3.7×10^{-2}
$MnSO_4\cdot4H_2O$	7.17×10^{-3}
$Co(NO_3)_2\cdot6H_2O$	1.72×10^{-3}
$ZnSO_4\cdot7H_2O$	1.56×10^{-2}
$CuSO_4\cdot5H_2O$	1.20×10^{-3}
$(NH_4)6Mo_7O_{24}\cdot4H_2O$	8.09×10^{-5}
Glucose	27.8
Hemin	1.00×10^{-2}

Table S2. All detected mutations in the chromosome, relative to the reference (NC_000911.1; Synechocystis sp. PCC 6803 chromosome, complete genome), by the genomic analysis. The variant frequency in the population of ancestral (Anc.), initially adaptive (Init-adapt.), and evolved cells (Evolved) are shown. ND indicates that mutations were not detected.

					Frequency			
	Position (nt)	Nucleotide changes	Gene	Strand	Anc.	Init-adapt.	Evolved	Notes
Single-base substitution	7438	T to C	photosystem II D1 protein	+	27%	27%	29%	
	7444	T to C	photosystem II D1 protein	+	28%	27%	29%	
	**335496**	**C to A**	**carboxysome formation protein CcmA**	**-**	**ND**	**ND**	**100%**	**Nonsynonymous: Gly to Glu @43**
	619733	A to G	probable esterase	+	100%	100%	100%	
	**829508**	**C to T**	**RNA polymerase alpha subunit**	**-**	**ND**	**ND**	**100%**	**Nonsynonymous: Arg to Gln @193**
	943495	G to A	P700 apoprotein subunit Ia	+	100%	100%	100%	
	1012958	G to T	hypothetical protein	-	100%	100%	100%	
	**1114921**	**G to C**	**periplasmic substrate-binding and integral membrane protein of the ABC-type Bgt permease for basic amino acids and glutamine BgtB**	**-**	**ND**	**ND**	**100%**	**Nonsynonymous: Phe to Leu @367**
	**1128135**	**C to G**	**unknown protein**	**-**	**ND**	**ND**	**100%**	**Nonsynonymous: Ala to Pro @625**
	1364187	A to G	orotidine 5' monophosphate decarboxylase	-	100%	100%	100%	
	1737000	C to A	similar to polyA polymerase	-	100%	100%	100%	
	1819782	A to G	photosystem II D1 protein	-	67%	66%	70%	
	1819788	A to G	photosystem II D1 protein	-	68%	68%	72%	
	2092571	A to T	asparaginase	-	100%	100%	100%	
	2198893	T to C	probable cation efflux system protein	-	100%	100%	100%	
	2301721	A to G	unknown protein	+	100%	100%	100%	
	2602717	C to A	unknown protein	+	100%	100%	100%	
	2602734	T to A	unknown protein	+	100%	100%	100%	
	2748897	C to T	two-component sensor histidine kinase	+	100%	100%	100%	
	3063738	G to A	two-component sensor histidine kinase	-	100%	100%	100%	
	3096187	T to C	putative transposase [ISY100v: 3095975 - 3096319, join 3097194 - 3097362, join 3098314 - 3098743]	+	70%	72%	61%	
	3110189	G to A	putative transposase [ISY523r: 3109761 - 3110626]	-	98%	100%	96%	
	3110343	G to T	putative transposase [ISY523r: 3109761 - 3110626]	-	88%	90%	94%	
	3142651	A to G	sucrose phosphate synthase	-	100%	100%	100%	
	**3203715**	**G to A**	**probable cation transporter**	**-**	**ND**	**ND**	**97%**	**Nonsynonymous: Pro to Ser @50**
Indel	**Next to 1905171**	**Del. GCCTCG**	**penicillin-binding protein**	**-**	**ND**	**ND**	**84%**	**In-frame deletion Ala-Glu @56-57**
	Next to 2204575	Del. G	a part of pilC, pilin biogenesis protein, required for twitching motility	+	81%	80%	81%	
	Next to 2350285	Ins. A	photosystem II reaction center PsbI protein	-	96%	97%	98%	
	Next to 2360245	Ins. C	hypothetical protein	+	95%	93%	93%	
	Next to 2409242	Del. C	unknown protein	-	95%	91%	98%	
	Next to 2419397	Del. T	hypothetical protein YCF22	-	99%	96%	98%	
	Next to 2544044	Ins. C	unknown protein	-	99%	99%	98%	
	Next to 2590063	Del. A	pilus biogenesis protein homologous to general secretion pathway protein E	+	93%	93%	95%	
	Next to 3260089	Del. C	hypothetical protein	-	76%	79%	82%	

Text S1

Here we derive Eq. 1 and 2 shown in the main text. We used an approximate continuous derivation for simplicity. We first assume a cell population with the frequency $h(z,t)$ in which each cell has a trait z (a variable that represents ln[IC_{50}]) and a specific growth rate $\mu(z)$, with a variable population size $N_h(t) = \int_{-\infty}^{\infty} h(z,t)dz$. When a cell with z produces an offspring, the trait of the progeny becomes $z+d_z$ with a probability r, or else becomes z (the same as the parent) $(1-r)$. Then, we derive the rate equation

$$\frac{\partial h(z,t)}{\partial t} = (1-r)\mu(z)h(z,t) + r\mu(z-d_z)h(z-d_z,t). \qquad \text{Eq. S1}$$

Here, $\mu(z)$ is described as $\mu(z)=\mu_{\max}e^z/(e^z+x)$ along with the main text definition ($\mu(z) = \mu_{\max}/(1+x/IC_{50}) = \mu_{\max}IC_{50}/(IC_{50}+x)$), where x is the toxic amino acid concentration. We approximated it as $\mu(z) \approx ce^z$, where $c = \mu_{\max}/x$, assuming $e^z+x \approx x$ in the transferred line during the experimental evolution. Note that the evolutionary properties with respect to generation (not time) do not depend on the absolute fitness (*i.e.*, c) but only depend on the relative fitness (c is canceled out below), and we ignored the fact that we changed the amino acid concentration x (thus c) in the experimental evolution.

Both terms on the right hand side are positive, with $0<r<1$ and $\mu(z)>0$, and the population size increases over time as

$$\frac{dN_h(t)}{dt} = \bar{\mu}(t)N_h(t), \qquad \text{Eq. S2}$$

derived by taking the integral of both sides of Eq. S1 with respect to z. Then, we derived the rate equation for the frequency $f(z,t)$ with the fixed population size N, *i.e.*, $f(z,t) = N \cdot h(z,t)/N_h(t)$, as

$$\frac{\partial f(z,t)}{\partial t} = (1-r)\mu(z)f(z,t) + r\mu(z-d_z)f(z-d_z,t) - \bar{\mu}(t)f(z,t), \qquad \text{(Eq. 1)}$$

from Eqs. S1 and S2. The generation is determined from the time variation in $N_h(t)$. From Eq. S2, $N_h(t)$ is solved as $N_h(t) = N_{h0}\exp[\int_0^t \bar{\mu}(t)dt]$, where N_{h0} is the total frequency at time 0. The generation g satisfies $N_h(t) = N_{h0}2^g$, and is solved as $g = \int_0^t \bar{\mu}(t)dt\,/ln2$. Thus

$$\frac{dg}{dt} = \frac{\bar{\mu}(t)}{\ln 2}, \qquad \text{Eq. S3}$$

which is required to obtain the evolutionary rate per generation (see below).

We roughly obtain an approximated analytical solution of the evolutionary rate of the mean of z (designated as M) per generation dM/dg ($=S_{IC50}$, when it is constant) in the model shown in Eq. 1. Because the trait z is a discrete variable with a step size d_z, we considered a short time period τ_M for the one-step change in M to approximately obtain dM/dg (using Eq. S3) as

$$\frac{dM}{dg}=\frac{dM}{dt}\frac{dt}{dg}=\frac{d_z}{\tau_M}\frac{\ln 2}{\bar{\mu}(t)}. \qquad \text{Eq. S4}$$

We assumed that M satisfies mode of $f(z,t)$ and $\mu(M)=\bar{\mu}$. The time period τ_M satisfies $f(M+d_z,t_0+\tau_M)=f(M,t_0)$ for a time t_0, assuming that the shape of the frequency distribution $f(M,t)$ does not change in this short time period. The frequency at $z=M+d_z$ can be solved as $f(M+d_z,t)=f(M+d_z,t_0)\exp[\{\mu(M+d_z)-\bar{\mu}\}(t-t_0)]$ from Eq. 1, by assuming that $\bar{\mu}$ is constant for this short time period and the contribution of r is negligibly small ($r\approx 0$). Then, τ_M satisfies $f(M+d_z,t_0)\,exp[\{\mu(M+d_z)-\bar{\mu}\}\tau_M]=f(M,t_0)$, and dM/dg can be solved from Eq. S4 as

$$\frac{dM}{dg}\approx 2(\ln 2)\sigma^2, \qquad \text{Eq. S5}$$

by assuming that $f(z,t)$ is a Gaussian with mean M and standard deviation σ and that $e^{d_z}-1\approx d_z$. Equation S5 means that the evolutionary rate is proportional to the variance (σ^2), known as Fisher's fundamental theorem of natural selection, and thus we should determine σ. We consider the one-step change of the edge ($z=z_E$) of the distribution because the edge and σ should be almost proportional (see below). We assume that z_E satisfies $f(z_E,t_0)=1$. Thus, $z_E\approx M+\sigma\sqrt{2\ln[N]}$, assuming that $f(z,t)$ is the Gaussian and $\ln[N]-\ln[\sigma\sqrt{2\pi}]\approx\ln[N]$. The time period for the one-step change at the edge (τ_E) is considered to satisfy $f(z_E+d_z,t_0+\tau_E)=1$. From Eq. 1, $f(z_E,t)=f(z_E,t_0)\,exp[\{\mu(z_E)-\bar{\mu}\}(t-t_0)]$ by assuming $r\approx 0$, and $f(z_E+d_z,t)=\int_{t0}^{t}r\mu(z_E)f(z_E,s)ds$ because $f(z_E+d_z,t_0)=0$. Thus, τ_E can be solved from the equation $\int_{t0}^{t0+\tau E}r\mu(z_E)f(z_E,t_0)\,exp[\{\mu(z_E)-\bar{\mu}\}(s-t_0)]\,ds=1$, and gives $dz_E/dg=(d_z/\tau_E)(ln2/\bar{\mu})$ as

$$\frac{dz_E}{dg}\approx\frac{(\ln 2)d_z\sigma\sqrt{2\ln N}}{\ln(1/r)}, \qquad \text{Eq. S6}$$

by assuming $1+r\approx 1$, $\ln[1/r]+\ln[1-e^{-\sigma\sqrt{2lnN}}]\approx\ln[1/r]$, and $e^{\sigma\sqrt{2lnN}}-1\approx\sigma\sqrt{2lnN}$. From Eqs. S5 and S6, the variation in σ ($\approx(ze-M)/\sqrt{2\ln[N]}$) is described as

$$\frac{d\sigma}{dg}\approx\ln 2\left(\frac{d_z}{\ln(1/r)}-\frac{2}{\sqrt{2\ln N}}\sigma\right)\sigma. \qquad \text{Eq. S7}$$

Eq. S7 means that σ becomes constant ($d\sigma/dg=0$) at

$$\sigma_{st}\approx\frac{d_z\sqrt{2\ln N}}{2\ln(1/r)}. \qquad \text{Eq. S8}$$

Thus, the evolutionary rate also becomes constant, and from Eqs. S5 and S8, it is described as

$$S_{IC50}\approx 2(\ln 2)\sigma_{st}^2=\ln 2\frac{d_z^{\,2}\ln N}{(\ln r)^2}. \qquad \text{(Eq. 2)}$$

This is the slope of the evolutionary change in $\ln[IC_{50}]$ per generation shown in Figure 3A-i.

Appendix S1. The source code in MATLAB for the numerical simulation that calculates the evolution shown in Fig. 3A-ii.

```
%parameter definitions
xmax=100;%max x (x=z/dz)
dz=0.4;%discrete step of z
r=0.00023;%probability
npop=10^7;%population size N
dNdt=0.01;%fraction of the increase in N for one calculation interval
grag=[20 40 60 80 100]; ndist=length(grag); pcol=jet(ndist);%for distribution graphs
gmax=100;%max generation for the calculation
tmax=round( gmax*log(2)/log(1+dNdt) );%max intervals for gmax
meanx=zeros(1,tmax);%mean of x
modex=zeros(1,tmax);%mode of x
edgex=zeros(1,tmax);%edge of x
varx=zeros(1,tmax);%variance of x
gen=zeros(1,tmax+1);%generation
f0=zeros(1,xmax);%frequency distribution
df=zeros(1,xmax);%change in frequency distribution
f1=zeros(1,xmax);%temporary variable for f0

%initial distribution
mean0=10; sd0=0;%mean and sd of x at time 0
f0 = exp(-(([1:xmax]-mean0).^2)./(2*(sd0^2)));%Gaussian if sd0~=0
if(sd0==0) f0=zeros(1,xmax); f0(mean0)=1.0; end%delta function if sd0=0
f0=npop*f0/sum(f0);%set the total population
f0=round(npop*f0/sum(f0));%for approximate discreet population

%evolution
c=0;%graph counter
for t=1:tmax;
    df(2:xmax-1) = (1-r)*exp(dz.*[2:xmax-1]).*f0(2:xmax-1) +
r*exp(dz.*[1:xmax-2]).*f0(1:xmax-2);%Eq.S1 (h -> f)
    f1=f0+df*( dNdt*sum(f0)/sum(df) ); % f1 = f0 + (df/dt)*dt;
    gen(t+1)=gen(t)+log2(sum(f1)/sum(f0));%calculation of generation
    f0=npop*f1/sum(f1);%dilution for constant population size
    f1 = f0 - f0.*(0<f0).*(f0<1)  +  ( (rand(1,xmax) < f0) .* ((0<f0).*(f0<1)) );%for
approximate discrete population
```

```
    f0=floor(f1);%approximate discrete population

    %distribution graphs
    if( (t>2) && ( gen(t-1)<=grag(c+1) ) && ( grag(c+1)<gen(t) ) );
        c=c+1;%graph counter
        subplot(4,1,1); plot(dz*[1:xmax],f0,'Color', pcol(c,:), 'LineStyle', '-'); axis([0
dz*xmax 0 max(f0)]); hold on %distribution graph
    end
    %representative values
    meanx(t)=sum([1:xmax].*f0)/sum(f0);%mean of x (=M/dz)
    modex(t)=max(find(max(f0)==f0));%mode of x
    edgex(t)=max(find(f0>0));%edge of x
    varx(t)=sum( ((([1:xmax]-meanx(t))).^2).*f0)/sum(f0);%var of x
end
gen(tmax+1)=[];%adjust vector size

%representative value graphs
subplot(4,1,2); plot([1:tmax],dz*(meanx-mean0),'.-'); axis([0 tmax
floor(dz*(meanx(2)-mean0)) ceil(dz*(meanx(tmax)-mean0))]); axis 'auto x';%mean of z (=M) -
meanz0 vs t
subplot(4,1,3); plot(gen,dz*(varx.^(0.5)),'b.',gen,dz*(edgex -
modex)/((2*log(npop))^0.5),'r-'); axis([0 gmax 0 1.2*dz*max(varx.^(0.5))]);%sd of z (=sigma)
vs g
subplot(4,1,4);
plot(gen,dz*(meanx-mean0),'b.',gen,dz*(modex-mean0),'r-',gen,dz*(edgex-mean0),'g-');
axis([0 gmax floor(dz*(meanx(2)-mean0)) ceil(dz*(meanx(tmax)-mean0))]); %mean(M) and edge(ze)
of z - meanz0 vs g

%approximate slope
poly=polyfit(gen,dz*meanx,1); [poly(1), 2*log(2)*(dz^2)*log(npop)/((log(r))^2)]%[regression
line, approximate analytical solution (Eq. 2)]
```
